# Supplementary material for: Effects of a peer educator program for HIV status disclosure and health system strengthening: Findings from a clinic-based disclosure support program in Mozambique
Source: PLoS One. 2020 May 6;15(5):e0232347. doi: 10.1371/journal.pone.0232347 (PMC7202645; doi:10.1371/journal.pone.0232347)
Supplement: Attachment — Submitted filename: PLos Queries and Repliescdr.3.28.docx [file pone.0232347.s001.docx]

1. Please ensure that your manuscript meets PLOS ONE's style requirements, including those for file naming. The PLOS ONE style templates can be found at

http://www.journals.plos.org/plosone/s/file?id=wjVg/PLOSOne_formatting_sample_main_body.pdf and <http://www.journals.plos.org/plosone/s/file?id=ba62/PLOSOne_formatting_sample_title_authors_affiliations.pdf>

*This has been done, thank you.*

2. Please include in your Methods section the date ranges over which you recruited participants to this study.

*On page 6 (‘Provider Training and Timelines’), we state when training and data collection occurred (2014).*

3. PLOS requires an ORCID iD for the corresponding author in Editorial Manager on papers submitted after December 6th, 2016. Please ensure that you have an ORCID iD and that it is validated in Editorial Manager. To do this, go to ‘Update my Information’ (in the upper left-hand corner of the main menu), and click on the Fetch/Validate link next to the ORCID field. This will take you to the ORCID site and allow you to create a new iD or authenticate a pre-existing iD in Editorial Manager. Please see the following video for instructions on linking an ORCID iD to your Editorial Manager account: <https://www.youtube.com/watch?v=_xcclfuvtxQ>

*This has been done, thank you.*

4. Please amend the manuscript submission data (via Edit Submission) to include authors Carol Dawson-Rose, Sarah Gutin, Florindo Mudender and Elsa Hunguana.

*This has been done, thank you.*

5. We note you have included tables to which you do not refer in the text of your manuscript. Please ensure that you refer to Tables 1, 2, 3 and 4 in your text; if accepted, production will need this reference to link the reader to each Table.

*All references now included, thanks.*

5. Review Comments to the Author

Reviewer #1: This paper describes the implementation and impact of a disclosure support intervention utilizing peer educators. This paper describes a unique intervention in a setting with exceptionally high rates of HIV and clear barriers around disclosure of HIV status. Overall, there appeared to be areas where the intervention could have been evaluated more thoroughly, which effected the overall impact of this study.

*Thank you and this is noted.*

I have the following suggestions for the submission:

Abstract

There were a lot of grammatical errors in the abstract throughout and would recommend the authors edit.

*This has been reviewed for grammar, thank you.*

Introduction

The introduction is well written overall and established the significance for this intervention in this setting. The authors use a lot of acronyms in this section and should ensure that they have always written out the meaning when first used (e.g. Paragraph 3, HCT).

*This has been reviewed and corrected, thank you. Of note, this phrase was fully spelled out in the first paragraph.*

Methods

Again, well written. However, there does not appear to be a lot of analysis around the feasibility or acceptability of this implemented intervention.

*The authors note that they did not measure feasibility and acceptability beyond (1) training, (2) procedures for the clinic, and (3) reports from PEs (Discussion).*

It was nice to hear how feedback from the peer educators was incorporated into the intervention, but no mention of whether this changed the number of PLWH who disclosed to their networks is mentioned in the study. This would also have been nice to conduct interviews with their contacts to see if they felt the cards helped in their ability to get tested.

*The authors agree that they do not have data on effectiveness of the intervention. Of note, there were not resources or time to collect data prior to PE implementation. Through our work with the PEs, this was identified, anecdotally, as a need. Of note, this is however included as a component of PDHP. Also of note, from the outset ,we had a positive response to the intervention implementation, and did not observe a related change in the absolute number of PLWH who reported disclosure, based on anecdotal information from PEs. These points have been included in the limitations section.*

Results

While there appears to be high utilization of this intervention, it is not clear whether the peer educators’ role in facilitating disclosure helped PLHIV or their partners get HIV testing or care.

*Because we did not have the capacity to track the denominators of those that recently disclosed, this issue presents a major limitation, and is noted in the limitations section. Further, we relied on data on reports that came to the health care facility for testing. We did not have human subjects approval to approach individuals in their home, or in the community -- only in the health facility.*

Discussion

The first paragraph mentions that this study cannot demonstrate that the intervention impacted community viral load, but this has been shown in other research. This may have been better placed in the introduction, framing the relevance of this type of intervention in Mozambique.

*This is now referenced in the second paragraph of the introduction, thank you.*

The second paragraph makes several points about the impact of the intervention that could be fleshed out.

*This has been done, thank you.*

Overall, this paper describes implementation of an intervention in a highly impacted and relevant setting, but lacks true evaluation around the acceptability and effectiveness of this intervention which diminishes its contribution to the field. This may be better presented in a shorter format, like a brief report.

*If acceptable, this has now been included in the limitations of the current format.*

Reviewer #2: The manuscript, "Effects of a peer educator program for HIV status disclosure and health system strengthening: Findings from a clinic-based disclosure support program in Mozambique" (PONE-D-19-29478) presents findings from a modestly sized, uncontrolled pilot study in which peer educators (PEs) meet with newly diagnosed HIV patients and encourage them to disclose to their partners. Strengths of the study include its use of PEs, rather than a highly skilled clinical professional, as well as the positive outcome of a large number of HIV positive persons being identified/seeking testing because of the disclosure process.

*Thank you for this.*

Despite the importance of this topic, significant flaws in this study undermine enthusiasm:

General

1. This might be a personal preference, but it appears the field is more commonly using "PLH" rather than "PLHIV."

*This has been changed throughout, thank you.*

2. From the abstract and throughout the manuscript, it is quite confusing that following ranged from "1 day to 3 months." Can the authors clarify this process?

*This was dependent on client availability, and has been clarified in the text.*

Introduction

1. The motivation for this study/intervention is somewhat muddled in the Intro. It appears that the priority is disclosure to partners but this is not made clear until the end of this section. Additionally, the focus relates to connecting additional individuals who might be HIV positive to testing and counseling. This, too, is somewhat unclear.

*This has been brought upwards in the introduction and edited for clarity.*

2. In contrast, very little is discussed about the benefits of disclosure to the PLH (e.g., social support, transportation to clinic, encouragement to take medication). This could be expanded or if it is not a priority, perhaps limit discussion of disclosure to family, friends, etc.

*This has now been included, thank you for this suggestion.*

3. Very little is said about the background related to peer counseling in the HIV field. Why is the use of PEs attractive? How would this contrast with other disclosure interventions?

*This has now been expanded on in the introduction section, thank you.*

Method

1. Who trained the PEs?

*I-TECH trainers who were expert trainers on PHDP conducted the training, and this has now been described in the manuscript (‘Provider Training and Timelines’).*

2. Was PE manualized? The results seem to suggest that PE was a 1-session intervention but no comments about the duration of PE are provided in the Method.

*The disclosure support is one component of the full training on dignity, positive health and prevention training. We adapted that component of the full training to a four-hour training for PE at the clinic. This is now also stated in the revised manuscript (‘Provider Training and Timelines’).*

Results

1. No specific outcome measures were used; therefore, it is difficult to interpret results related to disclosure (and connecting partners to testing/care). Did willingness to disclose change or improve with PE? This is unclear and severely undermines confidence that PE was specifically responsible for the disclosures.

*The is the same limitation as noted above from another reviewer, please see that response.*

2. Nearly 80% of partners who came in for testing were HIV positive. Did the PLH who underwent PE already know (or suspect) that their partner was positive? Could this relate to disclosure?

*The authors now recognize this in the limitations. The authors refer to Golden article (2017) on partner contact and tracing as being one of the most effective methods of HIV case finding. The authors recognize that our findings are not more broadly generalizable, as we report only on one health center. However, we can state that this approach was a new concept for PE at this facility, that had not been used previously.*

Discussion

1. The Limitations section significantly downplays the flaws in this study (and does not mention the biggest issues). Again, no measures were used so it is very difficult to determine if PE lead to a change in willingness/openness to disclose. Moreover, this was not a randomized trial. Therefore, it is impossible to determine if PE had an effect on disclosures.

*This has been addressed in an expanded version of the limitations section, thank you.*

Authors might want to consider focusing on feasibility and acceptability in a revision given the pilot nature of their work.

*This has now been more specifically included in the conclusions, highlighting that the pilot study also demonstrated feasibility and acceptability.*
